# Official Scientific Statement from the Brazilian Society of Cardiovascular Surgery - The 2021 ACC/AHA/SCAI Guideline for Coronary Artery Revascularization and the 2023 AHA/ACC/ACCP/ASPC/NLA/PCNA Guideline for Chronic Coronary Disease

**DOI:** 10.21470/1678-9741-2024-0990

**Published:** 2024-04-15

**Authors:** Nelson A. Hossne Jr, Luís Roberto Palma Dallan, Luiz Augusto Ferreira Lisboa, Henrique Murad, Walter José Gomes

**Affiliations:** 1 Cardiovascular Surgery Discipline, Hospital São Paulo, Escola Paulista de Medicina, Universidade Federal de São Paulo (UNIFESP), São Paulo, Brazil; 2 Cardiovascular Surgery Discipline, Instituto do Coração do Hospital das Clínicas da Faculdade de Medicina da Universidade de São Paulo (InCor-FMUSP), São Paulo, Brazil; 3 Universidade Federal do Rio de Janeiro (UFRJ), Rio de Janeiro, Brazil; 4 Academia Nacional de Medicina (ANM), Rio de Janeiro, Brazil

## INTRODUCTION

The American College of Cardiology (ACC)/American Heart Association (AHA) and the Society for Cardiac Angiography and Interventions (SCAI) published the 2021 Coronary Revascularization Guideline in January 2022 (approved by the respective committees in August 2021), replacing the 2011 Coronary Artery Bypass Graft and the 2011 and 2015 Percutaneous Coronary Intervention (PCI) guidelines^[1]^. Also, the Chronic Coronary Disease Guidelines was recently published, in August 2023, which “provides an update to and consolidates new evidence since the 2012 ACCF/ AHA/ACP/AATS/PCNA/SCAI/STS Guideline for the Diagnosis and Management of Patients With Stable Ischemic Heart Disease and the corresponding 2014 ACC/AHA/AATS/PCNA/SCAI/STS Focused Update of the Guideline for the Diagnosis and Management of Patients With Stable Ischemic Heart Disease (SIHD)”, stating that “A comprehensive literature search was conducted from September 2021 to May 2022.”^[2]^

Both documents stirred significant debate since their interpretation and recommendations were conflicted with the best available data, upsetting the global cardiovascular community managing patients with advanced coronary artery disease (CAD) and raising claims of inadequate evidence assessment and defective recommendations^[3,4]^.

In particular, unexpected changes in the recommendations of coronary artery bypass grafting (CABG) occurred related to left ventricular function and coronary anatomy setting. Specifically, the 2021 Guidelines for Coronary Revascularization downgraded CABG to improve survival compared with medical therapy from the 2011^[5]^ class of recommendation (COR) 1, level of evidence (LOE) B to COR 2b LOE B-R in patients with stable multivessel CAD and normal left ventricular ejection fraction (LVEF), and to COR 2a LOE B-NR in those patients with mild-to-moderate left ventricular dysfunction (LVEF 35%-50%). Furthermore, CABG was also downgraded from COR 2a LOE B^[5]^ to COR 2b LOE B-R in patients with stenosis in the proximal left anterior descending (LAD) artery. We will analyze and scrutinize the scientific data used to support the 2021 Guideline for Coronary Artery Revascularization and the 2023 Chronic Coronary Disease Guidelines, as well as compare them with previously published related guidelines.

Table 1 summarizes the timeline changes on CABG COR and LOE of the guidelines, according to the year of publication.

**Table 1:** Timeline changes on CABG class of recommendations and level of evidence of the guidelines, according to the year of publication.

Guidelines - Year of Publication (COR LOE)				
**Recommendations**	**1999^[6]^ and 2004^[7]^**	**2011^[5]^**	**2012^[8]^**	**2021^[1]^**
Multivessel^*^ normal EF	1 C Asymptomatic/Mild angina 1 A Stable angina	1 B	1 B	2a B-NR
Multivessel EF 35%-50%	1 C^‡^ Asymptomatic/Mild angina 1 A^§^ Stable angina	1 B	1 B	2a B-NR
Proximal LAD stenosis	2a A^**^	2a B	2a B	2b B-R

CABG=coronary artery bypass grafting; COR=class of recommendation; EF=ejection fraction; LAD=left anterior descending; LOE=level of evidence; R=randomized; NR=nonrandomized

The 2014^[6]^ Guidelines did not address CABG recommendations.

* Three-vessel disease with significant (≥ 70% diameter) stenosis in three major coronary arteries

‡ “Survival benefit is greater in patients with abnormal LV function, e.g., EF less than 0.50 and/or large areas of demonstrable myocardial ischemia.”^[6,7]^

§ “Survival benefit is greater when LVEF is less than 0.50.”^[6,7]^

** “This recommendation becomes Class 1 if extensive ischemia is documented by noninvasive study and/or LVEF is less than 0.50.”^[6,7]^

The authors of the 2021 guidelines have stated on the synopsis of recommendation-specific supporting text^[1]^ for downgrading CABG to COR 2b:

i. “The new Class 2b recommendation, which represents a downgrade from a Class 1 recommendation in the 2011 CABG guideline^[3]^, reflects new evidence showing no advantage of CABG over medical therapy alone to improve survival in patients with 3-vessel CAD with preserved LV function and no LM disease.” (...)
  (...) “Newer evidence from the ISCHEMIA trial^[7]^ and from metaanalyses, which incorporated^[8-11]^ or did not incorporate^[12]^ the ISCHEMIA results, as well as a more detailed review of earlier studies^[13]^ supported this downgrade.”

The only logical conclusion after the release of the 2021 and the revised 2023 guidelines is that new and compelling evidence in favor of medical treatment, when compared with CABG, was published in the meantime. Unfortunately, that is not the case. There isn’t a single published study, at any level, that was designed to directly address this comparison.

The 2023 Guidelines grade as COR 2a LOE B-R: “In patients with chronic coronary disease and multivessel CAD appropriate for either CABG or PCI, revascularization in addition to guidelinedirected management and therapy is reasonable to lower the risk of cardiovascular events such as spontaneous myocardial infarction, unplanned urgent revascularizations, or cardiac death.”^[2]^ Indeed, in the 2023 Chronic Coronary Disease Guidelines, the authors were given the opportunity to amend the previous distorted recommendations but instead opted for not colliding and even abstaining from specific contentious issues^[14]^.

In the seminal MASS II^[15]^ trial, the only trial ever comparing the strategies of optimal medical therapy (OMT), CABG, or PCI for multivessel CAD, proximal LAD CAD was present in 89% of patients in the OMT group, and in 93% of the CABG patients. Although the study design was insufficiently powered for assessing individual components of the composite endpoint, a significantly lower incidence of non-fatal myocardial infarction (MI) with CABG *vs.* OMT was seen at the 10-year (20.7 *vs.* 10.3, *P*=0.010) follow-up, but not at the five-year (*P*=0.785) follow-up. Cardiac death was significantly higher with OMT *vs.* CABG (20.7% *vs.* 10.8%, *P*=0.019), but at the five-year follow-up it was non-significant (12.3% *vs.* 7.9%, *P*=0.631). Overall mortality was reduced with CABG *vs.* OMT (25.1% *vs.* 31.0%, *P*=0.089), although not reaching statistical significance, at the five-year follow-up it was 12.8% *vs.* 16.2%, *P*=0.824. The pairwise comparison analysis showed a significant 2.02- and 2.77- fold increased risk of cardiac death and subsequent MI with OMT *vs.* CABG, respectively, demonstrating the progressively better long-term prognosis of surgical patients.

The guidelines authors’ assumption that the MASS-II trial may not represent the contemporary optimal medical treatment for CAD may be challenged by carefully reading the protocol and the serial publication of the one-year^[16]^, five-year^[17]^, and 10-year^[18]^ followups. All patients were placed on an optimal medical regimen at baseline until the end of follow-up, consisting of aspirin, β-blockers, angiotensin-converting enzyme inhibitors, calcium channel blockers, nitrates, and lipid-lowering agents, along with a low-fat diet, on an individual basis. All medications were dispensed free of charge to all patients throughout the 10-year follow-up to ensure protocol adherence.

The ISCHEMIA^[10]^ trial was not designed to compare CABG with OMT or PCI; therefore, inferences for recommendations based on the trial’s outcomes are not accurate in this sense. Patients were randomly assigned, including 5,179 patients with moderate or severe ischemia to an initial invasive strategy (angiography and revascularization when feasible) and OMT or to an initial conservative strategy of medical therapy alone and angiography if medicaltherapyfailed. Noteworthy, themethodofrevascularization in the invasive group, percutaneous or surgical, was not randomized. Among patients in the invasive-strategy group, 96% underwent angiography and 79.4% underwent revascularization (PCI in 74% and CABG in 26%), while in the medical therapy alone group, 21% underwent revascularization. Per protocol, CABG was recommended for participants with oneor two-vessel disease only if they had severe diffuse disease, significant calcification or vessel tortuosity, complex branch lesions, complex chronic total occlusions supplying viable myocardium, or situations otherwise unfavorable for PCI. For participants with three-vessel disease (3VD), the SYNTAX score guided the selection of the invasive procedure. PCI was recommended for low SYNTAX scores (0 to 22), CABG was recommended for high scores ≥ 33, and PCI or CABG could be performed for intermediate SYNTAX scores (23 to 32). In patients with diabetes mellitus, PCI was recommended to treat only non-complex focal or discrete atherosclerotic disease. In synthesis, CABG was performed in a population of more advanced CAD and PCI in a group of lower-risk patients. Notwithstanding, the ISCHEMIA trial grouped and analyzed CABG and PCI as a single revascularization group (invasive strategy group).

We must point out and stress that grouping CABG and PCI as an equivalent revascularization strategy for comparative analysis is somewhat concerning since they are complementary procedures with mostly different indications and outcomes, and no evidence supports this approach.

The BARI-2D^[18]^ study demonstrated that a strategy of prompt coronary revascularization in patients who had been treated with intensive medical therapy for diabetes and stable ischemic disease did not significantly reduce the rate of death from any cause or major cardiovascular events. However, among patients for whom CABG was deemed the appropriate treatment, prompt revascularization reduced the rate of major cardiovascular events compared with medical therapy, particularly among patients assigned to receive insulin sensitization (*P*=0.002). In the PCI stratum, however, revascularization did not reduce the rate of death or major cardiovascular events when added to medical therapy.

The COURAGE^[19]^ trial, a multicenter study, randomized 2287 patients who had objective evidence of myocardial ischemia and significant CAD to undergo PCI with OMT or OMT alone, with a median follow-up period of 4.6 years. “There were no significant differences between the PCI group and the medical-therapy group in the composite of death, myocardial infarction, and stroke (20.0% *vs.* 19.5%; hazard ratio, 1.05; 95% CI, 0.87 to 1.27; *P*=0.62); hospitalization for acute coronary syndrome (12.4% *vs.* 11.8%; hazard ratio, 1.07; 95% CI, 0.84 to 1.37; *P*=0.56); or myocardial infarction (13.2% *vs.* 12.3%; hazard ratio, 1.13; 95% CI, 0.89 to 1.43; *P*=0.33).”

The REVIVED-BCIS2^[20]^ trial randomized 700 patients with a LVEF ≤ 35%, extensive CAD amenable to PCI, and demonstrable myocardial viability to a strategy of either PCI plus OMT (PCI group) or OMT alone (OMT group). The findings after a median of 3.4 years revealed that among patients with severe ischemic left ventricular systolic dysfunction who received OMT, revascularization by PCI did not result in a lower incidence of death from any cause or hospitalization for heart failure (hazard ratio, 0.99; 95% confidence interval [CI], 0.78 to 1.27; *P*=0.96). Additionally, no incremental improvement in the LVEF or a sustained difference in quality of life was seen.

In the SYNTAX trial (1800 patients randomly assigned to the PCI [n=903] or CABG [n=897]), one-year^[21]^, five-year^[22]^, and 10-year^[23]^ follow-ups consistently demonstrated that “CABG remains the standard of care for patients with three-vessel CAD”, by providing a significant survival benefit.

As a matter of fact, the MASS^[16]^, MASS II^[18]^, SYNTAX^[22-24]^, STICH^[24]^, and FREEDOM^[25]^ trials have shown increasing benefits for patients who underwent CABG at a longer follow-up, beyond five years.

Additionally, the FAME 3^[26]^ trial, an ongoing multicenter, international, noninferiority trial, was published in 2021 (after the 2021 guideline writing committee approved the guidelines) and randomized 1,424 patients with three-vessel CAD to undergo CABG or fractional flow reserve (FFR)-guided PCI with currentgeneration zotarolimus-eluting stents. Nearly 82% of patients had preserved left ventricular function. The primary endpoint was the occurrence within one year of a major adverse cardiac or cerebrovascular event, defined as death from any cause, MI, stroke, or repeat revascularization. The conclusion revealed that in patients with three-vessel CAD, FFR-guided PCI was not found to be non-inferior to CABG concerning the incidence of major adverse cardiac or cerebrovascular event, defined as a composite of death, myocardial infarction, stroke, or repeat revascularization at one year. Besides, the 30-day mortality rate in the CABG arm was 0.3%, identical to that of PCI, demonstrating indisputable advances in contemporary surgical techniques.

The three-year^[27]^ follow-up of the FAME 3 trial, published after the 2021 and 2023 guidelines, showed a broadening divergence between PCI and CABG outcomes on the primary endpoint, 18.6% *vs.* 12.5%; *P*=0.002, with the difference between the incidence of MI becoming statistically significant, respectively 7.0% *vs.* 4.2%, *P*=0.02. Concerning the key secondary outcome of death, MI, or stroke at three years, the FAME 3 three-year follow-up showed a difference with 9.2% in the CABG group and 12.0% in the PCI; *P*=0,07, widening the gap obtained at 1-year follow-up with 5.2% *vs.* 7.3%, respectively. However, the conclusion of the FAME-3 threeyear trial focused on the results of the non-statistically powered key secondary outcome rather than the primary endpoint.

Findings from the FAME-3 three-year follow-up are aligned with the SYNTAX trial; the incremental benefit of surgery was greater the higher the SYNTAX score, the severity extent, and the more diffuse the coronary disease. For patients with a low functional SYNTAX score (≤ 22), the numerical advantage of CABG *vs.* PCI was not statistically significant. For patients with a high functional SYNTAX score (> 22), the incremental benefit of surgery is highly statistically significant, and these differences continue to widen over time. Thus, reinforcing that surgery is superior in patients with a functional SYNTAX score (> 22). Longer follow-up is imperative to assess these data, as previously published studies demonstrated that CABG, when compared to PCI, provides a long- _term benefit_[16,18,22-26]_._

Recurrently, strong evidence reinforces that PCI does little to protect coronary circulation from plaque rupture and consequent MI, the harbinger of the outcomes of death and heart failure in CAD. As the COURAGE, MASS-II, BARI 2D, and REVIVED trials thoroughly demonstrated, PCI does not reduce rates of spontaneous MI and long-term mortality in patients with stable disease compared to OMT. In contrast, CABG does reduce the long-term rates of MI and mortality in advanced CAD.

Since the aforementioned trials demonstrated CABG is superior to PCI in these patients, would it be reasonable to assume PCI is inferior to medical treatment, so as to explain the lack of benefit in studies that compared revascularization with medical treatment, but grouped CABG and PCI in a single revascularization arm?

The 2021 Guidelines stated CABG is superior to PCI in survival, regardless of the ejection fraction in patients with 3VD, and that “CABG may be reasonable to improve survival” (COR 2b LOE B-R) in multivessel CAD patients with normal ejection fraction. Conversely, the same recommendation (COR 2b LOE B-R) was given to PCI in these patients, while disclosing “the usefulness of PCI to improve survival is uncertain”. Could it also be inferred that PCI is inferior to medical treatment? These two side-by-side recommendations are contradictory and conflicting per se.

Aside from the ISCHEMIA trial, the following evidence from metaanalysis comparing revascularization with medical therapy was referenced to support the 2021 guidelines.

Bangalore et al.^[11]^ analyzed 14 randomized trials that enrolled 14,877 patients. “Most trials enrolled patients with preserved left ventricular systolic function and low symptom burden and excluded patients with left main disease. Revascularization compared with medical therapy alone was not associated with a reduced risk of death (relative risk, 0.99 [95% CI, 0.90-1.09]).” Identical to the ISCHEMIA trial, PCI and CABG were grouped together in the revascularization arm, and out of the 14,877 patients, approximately 4,692 patients received CABG (only 32.6%), reinforcing that most of the weight of this meta-analysis results came from PCI *vs.* medical treatment.

Vij et al.^[13]^ enrolled seven randomized controlled trials, analyzing a total of 12,013 patients - PCI and CABG were analyzed as a single group. The authors stated: “Although CABG and PCI are acceptable means of coronary revascularization, the trials included in our meta-analysis have PCI as the predominant means of revascularization, except for BARI-2D, MASS-II and ISCHEMIA trial where a significant proportion (32%, 33%, and 26%, respectively) of patients underwent CABG.” Once more, an overwhelming proportion of patients underwent PCI, and not CABG, as revascularization strategy.

Laukkanen et al.^[14]^ extracted data from 14 randomized trials, comprising of 15,774 patients. “There was no significant difference in all-cause mortality risk (0.95, 95% CI: 0.86-1.06); however, revascularization plus medical therapy reduced the risk of the composite outcome of all-cause mortality, myocardial infarction, revascularizations, rehospitalizations, or stroke (0.69, 95% CI: 0.55-0.87); unplanned revascularization (0.53, 95% CI: 0.40-0.71); and fatal myocardial infarction (0.65, 95% CI: 0.49-0.84).” Again, PCI and CABG were enrolled in the same arm. Out of the 15,744 patients, approximately 2,307 patients received CABG (barely 14.7%).

These studies were not designed to compare CABG with medical treatment, nor had the power to do so, all of which had a major confounding factor such as the enrollment of patients who underwent PCI or CABG in a single revascularization arm - with the major caveat that most patients in these trials underwent PCI. Navarese et al.^[12]^ published a meta-analysis of cardiac mortality in patients randomized to elective coronary revascularization plus medical therapy or medical therapy alone, comprising of 25 trials and 19,806 patients. Interestingly, the results were contradictory to the 2021 guidelines recommendations: “Compared with medical therapy alone, revascularization yielded a lower risk of cardiac death [RR 0.79 (0.67-0.93), *P*<0.01] and spontaneous MI [RR 0.74 (0.64-0.86), *P*<0.01]. By meta-regression, the cardiac death risk reduction after revascularization, compared with medical therapy alone, was linearly associated with follow-up duration [RR per 4-year follow-up: 0.81 (0.69-0.96), *P*=0.008], spontaneous MI absolute difference (*P*=0.01) and percentage of multivessel disease at baseline (*P*=0.004)”. The authors concluded that “The present large-scale analysis of randomized trials shows a significant and consistent reduction of cardiac mortality in favor of elective coronary revascularization plus medical therapy compared with medical therapy alone in stable CAD patients, the magnitude of which is directly associated with duration of follow-up and a lower risk of spontaneous MI”.

The only meta-analysis that did not incorporate the ISCHEMIA trial, published by Windecker et al.^[15]^, included 100 trials in 93,553 patients with 262,090 patient years of follow-up. The results were clearly in favor of CABG, which “was associated with a survival benefit (rate ratio 0.80, 95% credibility interval 0.70 to 0.91) compared with medical treatment”. The conclusion of this meta-analysis stated that “Among patients with stable coronary artery disease, coronary artery bypass grafting reduces the risk of death, myocardial infarction, and subsequent revascularization compared with medical treatment.”, which is incompatible with the guideline’s recommendations.

Given the contradictory evidence used to support the recommendations, the total absence of new data and the overwhelming number of studies demonstrating CABG superiority against either PCI or medical treatment, the decision of the writing committee of the 2021 guideline to impactfully downgrade CABG is uncomprehensive and misleading.

ii. “The older recommendation was based on evidence from registry studies^[28-31]^, a meta-analysis^[32]^, and a single RCT^[33]^, all of which were completed >20 to 40 years ago before the development of newer surgical techniques or advances in medical therapy associated with improved prognosis^[34,35]^.”

Disregarding evidence solely based on date of publication, without any specific new data to back up your conclusion, is a logical fallacy. Surprisingly, only evidence to highlight advances in medical therapy - antiplatelet and statin - is referenced here, albeit surgical developments in off-pump and aorta no-touch procedures, multiarterial grafting, epiaortic scanning, intraoperative graft assessment, minimally invasive procedures, among others are largely documented^[36,37]^.

On top of surgical advances, newer publications have reinforced the superiority of CABG compared to medical treatment.

Gaudino^[38]^ et al. published, in 2022, an individual patient data pooled meta-analysis of four randomized trials comparing CABG with medical treatment, including 2,523 patients with a median follow-up of 5.6 years. “The cumulative 10-year mortality rate was lower in patients treated with CABG compared with MT (45.1% *vs.* 51.7%, respectively; odds ratio, 0.70; 95% CI, 0.58-0.85). In patients with stable CAD, initial allocation to CABG was associated with greater periprocedural risk of death but improved long-term survival compared with MT. The survival advantage for CABG became significant after the fourth postoperative year.”

Galli^[39]^ et al. performed a meta-analysis, published in 2023 (available online since October 2022), including 18 randomized controlled trials comparing different revascularization strategies - angiography-guided PCI, physiology-guided PCI, and CABG - in 26,625 patients with CAD without left-main disease or reduced LVEF, with a mean follow-up of 5.1 years. “CABG was the only revascularization strategy associated with reduced cardiovascular death (IRR 0.76, 95% CI 0.64-0.89) and all-cause death (IRR 0.87, 95% CI 0.77-0.99), compared with medical therapy.”

iii. “After several hours of deliberation, the writing committee concluded that using CABG as a revascularization strategy versus medical therapy alone “may be reasonable” to improve survival in stable patients with 3-vessel CAD. The writing committee recognized that an adequately powered trial to test this hypothesis is unfeasible in the current era but proposed that revascularization confers other benefits to patients with multivessel CAD and SIHD.”

After several hours of deliberation, the Society of Thoracic Surgeons/American Association for Thoracic Surgery (STS/AATS) unprecedently decided to withdraw the endorsement of the 2021 ACC/AHA/SCAI Guideline for Coronary Artery Revascularization. The Heart Team, especially for multivessel disease, was unambiguously dissolved, going against consecutive guidelines own COR 1 LOE B-NR.

The STS/AATS-endorsed rebuttal to 2021 ACC/AHA/SCAI Chronic Coronary Disease Guideline was the only reasonable option available, yet unfortunate, given the utmost negative repercussions of a published guideline without the endorsement of all related Societies. We can only skim the surface of the major drawbacks involved in such decision, given it directly addresses a disease - and its treatments - with the largest worldwide impact in mortality and health burden-related costs. It ultimately leads, at best, to unsafety and uncertainty for patients, healthcare professionals, and health insurance companies.

The underlying reason for the final approval and subsequent release of the 2021 ACC/AHA/SCAI Coronary Artery Disease Guidelines - and its 2023 revision -, in which CABG stands as the recommended treatment in selected groups of patients, after the unprecedented rebuttal of the STS/AATS, is certainly beyond the scope of a scientific statement, albeit a major failure. At least, the guidelines publication should had been postponed until an adequate and appropriate consensus or agreement had been fulfilled.

One of the main scopes of any guideline is to provide a guidance which must account for the best evidence-based treatments available, resulting in unequivocal patient protection. Obviously, medicine is a constant evolving field, but any changes in direction, particularly those that carries a worldwide impact in medical practice, must be supported by new published evidence.

Guidelines are not mandatory in nature, nor have the power to impose treatment decision making. Nevertheless, it is - or should be - an unbiased guidance to the best evidence derived treatment available, with all the concerns and legal issues that may arise whether one would choose to pursue a different path. Therefore, guidelines publication carries a strong scientific weight, for which authors’ responsibility must be accounted.

Shouldn’t we expect or should we even need to ask for an unbiased, balanced, accurate, and comprehensive guideline? The inaccuracy and evidence misinterpretation of the 2021 ACC/ AHA/SCAI Coronary Guidelines shouldn’t have happened in the first place. Reaching a consensus endorsed by different Societies is not an easy task, but it is even harder to convince the scientific community after all these controversies and surrounding debates that previously published guidelines were reliable and solely focused on patients’ best interests and care. Credibility is simply the standard by which guidelines and, hence, medical societies are measured. This question ought not be taken as a harsh and/ or direct criticism towards any specific Society, but rather a call for reflection to all Societies historically involved in the publication of coronary revascularization guidelines. It is the only reasonable option and viable path that should be taken to ensure and strength what physicians must always embrace: “(…) Into whatever homes I go, I will enter them for the benefit of the sick (…)”^[40]^.

## Abbreviations, Acronyms & Symbols

AATS: American Association for Thoracic Surgery
ACC: American College of Cardiology
AHA: American Heart Association
CABG: Coronary artery bypass grafting
CAD: Coronary artery disease
CI: Confidence interval
COR: Class of recommendation
EF: Ejection fraction
FFR: Fractional flow reserve
LAD: Left anterior descending
LOE: Level of evidence
LVEF: Left ventricular ejection fraction
MI: Myocardial infarction
OMT: Optimal medical therapy
PCI: Percutaneous coronary intervention
SCAI: Society for Cardiac Angiography and Interventions
SIHD: Stable Ischemic Heart Disease
STS: Society of Thoracic Surgeons
3VD: Three-vessel disease
